# Are there changes in medical specialist contacts after transition to a nursing home? an analysis of German claims data

**DOI:** 10.1186/s12913-020-05575-x

**Published:** 2020-08-04

**Authors:** Ove Spreckelsen, Guido Schmiemann, Michael H. Freitag, Alexander M. Fassmer, Bettina Engel, Falk Hoffmann

**Affiliations:** 1 Department of Health Services Research, Division of General Practice, Carl von Ossietzky University of Oldenburg, 26111 Oldenburg, Germany; 2 Department of Health Services Research, Institute for Public Health and Nursing Science, University of Bremen, Bremen, Germany; 3 Institute for General Practice, Hannover Medical School, Hannover, Germany; 4 Department of Health Services Research, Division of Outpatient Care and Pharmacoepidemiology, Carl von Ossietzky University of Oldenburg, Oldenburg, Germany

**Keywords:** Nursing home residents, Primary care, Specialist care, Health services research, Secondary data analysis, Germany

## Abstract

**Background:**

Provision of ambulatory care by medical specialists for nursing home residents (NHR) is discussed to be inadequate in Germany, however with only incomplete evidence on this topic. We wanted to know whether the transition to a nursing home is associated with a general decrease in medical specialist care and therefore compared contact rates before and after institutionalization.

**Methods:**

Claims data of 18,779 newly admitted NHR in 2013 were followed for the whole year prior to and up to two years after admission. The frequencies of contacts to specialists were assessed and stratified by sex, age, care level, dementia diagnosis and chronic conditions. Multivariate analyses were conducted to identify predictors for contacts to specialists.

**Results:**

One year after institutionalization the most pronounced decrease was found in contacts with ophthalmologists (38.4% vs. 30.6%) whereas with most other specialties only small changes were found. The only specialty with a large increase were neurologists and psychiatrists (27.2% vs. 43.0%). Differences depending on sex and age were rather small while NHR with dementia or a higher care level had lower contact rates after institutionalization. Before institutionalization most patients were referred to a specialist by a general practitioner (61.7–73.9%) while thereafter this proportion decreased substantially (27.8–58.6%). The strongest predictor for a specialist contact after admission to a nursing home was a contact to a specialist before (OR 8.8, CI 7.96–9.72 for contacts to neurologists or psychiatrists). A higher nursing care level and a higher age were also predictors for specialist contacts.

**Conclusions:**

Relevant decreases of ambulatory specialist care utilization after institutionalization are restricted to ophthalmologists. NHR of higher age and higher nursing care level had a lower chance for a specialist contact. The assessment of the adequacy of the provided care after institutionalization remains inconclusive due to little investigated but assumable changes in care needs of NHR. The decreased coordination of care by general practitioners after institutionalization conflicts with health policy goals.

## Background

In Germany, about 818,000 persons are living in 11,200 nursing homes [1]. Nursing home residents (NHR) are a population with a high disease burden, multimorbidity, mobility constraints and cognitive deficits [2–4]. Malnutrition [5], oral health problems [6] or visual impairments and hearing loss [7] represent specific health problems. Also, a number of medical care problems for NHR are being discussed in health services research and policy: high prescription rates of psychotropics [3, 8], inadequate medication use in renal insufficiency [9], or lower influenza vaccination rates than recommended by guidelines [10]. These health issues also affect the use of different healthcare sectors and medical specialists. For example, a part of the hospitalizations of NHR is discussed as potentially avoidable [11]. There is evidence for the inadequate provision of eye-care [12], preventative services [13] or palliative care [14] in nursing homes. In Germany, too few ophthalmological control examinations of NHR with diabetes mellitus and glaucoma [15] or inadequately low contact rates to dentists are equally problematized [16]. Compared to the general population of the same age-groups, lower utilization rates of medical specialists by NHR with the exception of neurologists and psychiatrists were found [2]. Lower utilization rates by older NHR, those with dementia and with a higher care level are further seen as indicators of less than optimal care [3].

Furthermore, NHR - as any other citizen in Germany - can directly access medical specialists in the ambulatory sector since no gatekeeping system of general practitioners (GPs) is established on an mandatory basis [17]. Transportation of a NHR with mobility constrains to a medical practice is usually covered by the health insurance, but due to the high prevalence of more severe mobility constraints home visits play an important role for health care in the nursing home population. Medical care in nursing homes is mainly provided by GPs [3, 18, 19] and they more often conduct home visits than medical specialists for NHR [20]. However, the degree of GP coordination of specialist contacts is not known. Overall, only limited data is available for the utilization of medical specialists of NHR in Germany [8]. Information on this topic is based on surveys among nurses [20, 21], cross-sectional studies in conveniently sampled nursing homes [18, 19] or comparisons of NHR and the non-institutionalized population of the same age-groups with claims data [2, 22]. Therefore, most of these studies did not assess whether institutionalization itself has an influence on contact rates to medical specialists. French analyses with reimbursement data show that nursing home admission is associated with decreases in contacts to specialists [23]. International evidence is limited and to our knowledge there are no studies from Germany providing a comprehensive view on differences in specialist contact rates and GP referrals before and after admission to a nursing home.

The aim of this study is therefore to describe differences in contact rates with medical specialists and the extent of GP-coordination before and after institutionalization.

## Methods

### Database and study population

Claims data of the DAK-Gesundheit, which is one of the largest German health insurance funds operating nationwide (6 million members; 7.5% of the German population), were analyzed. We included all persons aged 65 years and older, who were newly admitted to a nursing home between January 1 and December 31, 2013 and followed them for up to 2 years. New admission was defined by the preceding period of at least 365 days in which the person was continuously insured and did not yet live in a nursing home. To assure a comparable time at risk, residents had to be insured at least 1 day in each of the four quarters after admission. That also includes those who died within the last quarter after admission but ensures that all NHR had to be alive and therefore “at risk” for physician contacts for three quarters after admission. For the sensitivity analyses a subgroup was built including only residents who were insured at least 1 day in each of the following eight quarters after institutionalization (e.g. including those that died within the eighth quarter after admission).

Information on the date of institutionalization was gathered from the German Long-Term Care Insurance (“Gesetzliche Pflegeversicherung”). These data also include levels of care dependency. During the years of observation, there were three care levels ranging from considerable need of care (level I) to most heavily care dependent (level III). Further information on the German long-term care system can be found elsewhere [17]. In principle, all residents are assigned to a care level on the day of admission. In case of an increase in care dependency care levels can be reevaluated and adapted.

We further used data on reimbursement of physicians working in ambulatory care, including both contacts in their own practice as well as visits in the corresponding nursing home. Information include diagnoses according to the German modification of the International Classification of Diseases (ICD-10 GM) and whether a patient was referred by a GP. Data are limited to the information whether a contact has taken place in a quarter at all and hold no information of specific dates, or number of contacts.

### Outcome and independent variables

Our outcomes of interest were at least one contact with a physician of each of the specialties and with a GP. This was assessed both for the year before and the year after admission to a nursing home (e.g. the four quarters before as well as after the admission to a nursing home). We restricted the analyses to the information whether a contact happened at all in that year as reimbursement data do not contain the information of the exact number of contacts within a time period. As independent variables we assessed age (65–74, 75–84, 85–94 and 95+ years), sex (males and females), and care levels (3 levels). Care levels were obtained on the day of institutionalization and serve as a proxy for functional status. The following indicator diagnoses were assessed in the year before institutionalization in ambulatory care: diabetes mellitus, glaucoma, depression, dementia, Parkinson’s disease and prostate hyperplasia. We chose those diagnoses as they represent common health problems among NHR, are regular reasons to involve medical specialists or due to the availability of guideline recommendations about the frequency of specialist contacts [24–26].

The most common specialist groups according to the number of practices in ambulatory care were assessed, namely, dentists, ophthalmologists, surgeons, gynecologists, ear nose throat (ENT)-specialists, dermatologists, urologists, and neurologists and psychiatrists. Additionally, we assessed contacts to GPs.

### Statistical analysis

We conducted comparisons of the proportions of at least one contact to a physician between the year before and after institutionalization for every medical specialty and GPs separately (i.e. whether a contact took place at all in the two observational periods). These analyses were stratified by sex, age group, and care level. Furthermore, we compared the difference in contact rates to the subsequent specialists by the above-mentioned indicator diagnoses. Multivariate analyses were conducted to assess predictors for a specialist contact in the year after admission to a nursing home and were presented as odds ratios (ORs) with 95% confidence intervals (95% CI). We included age (65–74, 75–84, 85–94 and 95+ years), sex (males and females), care levels (3 levels) as well as dementia (yes vs. no) and specialists contact in the year before admission (yes vs. no) as independent variables.

Coordination of care was assessed by contacts to specialists with a documented referral by a GP. Therefore, for all NHR with at least one contact to ophthalmologists, surgeons, gynecologists, ENT specialists, dermatologists, urologists, and neurologists and psychiatrists in the year before and after institutionalization, we assessed the proportion being referred by a GP. Thus, denominators of those analyses only include persons with at least one specialist contact within the corresponding year.

In the sensitivity analysis conducted within the subgroup who were insured at least 1 day in each of the eight quarters after institutionalization, we also compared the proportion of residents that were seen by a GP and by a medical specialist at least once in the year before as well as in the first and second year after institutionalization.

All statistical analyses were performed with SAS for Windows version 9.4 (SAS Institute Inc., Cary, North Carolina).

## Results

### Baseline characteristics

In total 18,779 persons were institutionalized in 2013 and were alive up to the fourth quarter after admission (Table 1). The average age was 84.2 years, 77.0% were female. Most participants were assigned to the lowest care level (66.4%). At the beginning of the study period, 37.0% were diagnosed with dementia.

**Table 1:** Baseline characteristics of the study population

	Total	Male	Female
	*n* = 18,779	*n* = 4326 (23.0%)	*n* = 14,453 (77.0%)
**Age in years**			
Ø (SD)	84.2 (7.1)	82.5 (7.3)	84.7 (6.9)
65–74	11.0% (*n* = 2073)	16.1% (*n* = 697)	9.5% (*n* = 1376)
75–84	34.7% (*n* = 6519)	39,4% (*n* = 1704)	33,3% (*n* = 4815)
85–94	50.6% (*n* = 9493)	42.0% (*n* = 1816)	53.1% (*n* = 7677)
95+	3.7% (*n* = 694)	2.5% (*n* = 109)	4.0% (*n* = 585)
**Care level**			
1	66.4% (*n* = 12,470)	58.9% (*n* = 2549)	68.6% (*n* = 9921)
2	29.4% (*n* = 5529)	35.6% (*n* = 1539)	27.6% (*n* = 3990)
3	4.2% (*n* = 780)	5.5% (*n* = 238)	3.8% (*n* = 542)
**Death within study period**			
Yes	20.2% (*n* = 3784)	26.9% (*n* = 1165)	18.1% (*n* = 2619)
No	79.8% (*n* = 14,995)	73.1% (*n* = 3161)	81.9% (*n* = 11,834)
**Disease prevalence**			
Diabetes	31.7% (*n* = 5954)	37.9% (*n* = 1639)	29.9% (*n* = 4315)
Glaucoma	14.0% (*n* = 2631)	12.7% (*n* = 549)	14.4% (*n* = 2082)
Depression	31.2% (*n* = 5863)	23.1% (*n* = 999)	33.7% (*n* = 4864)
Dementia	37.0% (*n* = 6951)	39.5% (*n* = 1708)	36.3% (*n* = 5243)
Parkinson’s disease	7.7% (*n* = 1445)	11.9% (*n* = 514)	6.4% (*n* = 931)
Prostate hyperplasia	N/A	38.6% (*n* = 1670)	N/A

### GP and specialist contacts before and after institutionalization

A very high percentage (96.8%) of the participants were seen at least once a year by a GP prior to institutionalization, thereafter almost all (99.6%).

Compared to the year prior to institutionalization different trends appeared for different specialties (Table 2). The largest decrease in contact rates was found in eye care where fewer residents were seen by an ophthalmologist compared to the year before admission (38.4% vs. 30.6%). Smaller decreases were found among surgeons and gynecologists. For all other specialties contact rates increased. The largest increase in the year after admission was seen for contacts with neurologists and psychiatrists (27.2% vs. 43.0%). Other specialties with smaller increases were dentists, ENT specialists, dermatologists and urologists.

**Table 2:** Proportion of specialist contacts (and the proportion of those being referred by a GP) before and after institutionalization (*n* = 18,779)

	One year before	One year after
Dentist	44.6%	50.4%
Ophthalmologist	38.4% (69.0%)	30.6% (34.1%)
Surgeon	30.2% (70.7%)	26.6% (53.9%)
Gynecologist (women only)	9.6% (61.7%)	7.5% (27.8%)
ENT specialist	21.0% (63.1%)	25.6% (30.6%)
Dermatologist	15.8% (68.3%)	17.9% (39.3%)
Urologist	13.8% (71.4%)	17.1% (50.0%)
Neurologist/Psychiatrist	27.2% (73.9%)	43.0% (58.6%)

**Abbreviation:***ENT* Ear, nose and throat

Referrals from GPs to dentists were not assessed since those are very unusual in the German health care system

### Patterns by age, sex, care levels and indicator diagnoses

As patterns by sex, age and care level were comparable before and after institutionalization (Table 3), only the latter ones are reported. Large sex differences appeared only with contacts to urologists (men 39.2% vs. 10.4% women). Differences found in other specialties were small.

**Table 3:** Proportion of specialist contacts by sex, age and care level before and after institutionalization (*n* = 18,779)

			One year before	One year after
Dentist	Sex	Women	43.4%	49.8%
		Men	48.9%	52.1%
	Age group	65–74 y	46.7%	53.6%
		75–84 y	49.8%	53.9%
		85–94 y	41.3%	45.7%
		95+ y	35.0%	43.8%
	Care level	1	45.0%	51.1%
		2	43.8%	49.5%
		3	44.4%	44.6%
Ophthalmologist	Sex	Women	38.5%	30.9%
		Men	38.3%	29.7%
	Age group	65–74 y	29.2%	26.7%
		75–84 y	39.9%	30.4%
		85–94 y	39.9%	31.8%
		95+ y	32.4%	27.2%
	Care level	1	40.4%	33.8%
		2	35.3%	25.4%
		3	29.2%	17.2%
Surgeon	Sex	Women	31.0%	27.5%
		Men	27.5%	23.6%
	Age group	65–74 y	31.1%	27.7%
		75–84 y	34.4%	28.6%
		85–94 y	27.8%	25.3%
		95+ y	20.6%	22.2%
	Care level	1	31.9%	28.5%
		2	27.4%	23.7%
		3	22.4%	16.4%
Gynecologist (women only)	Age group	65–74 y	13.3%	9.6%
		75–84 y	12.1%	8.3%
		85–94 y	7.4%	6.6%
		95+ y	4.5%	4.3%
	Care level	1	10.1%	7.8%
		2	8.5%	7.1%
		3	9.2%	5.3%
ENT specialist	Sex	Women	20.6%	26.0%
		Men	22.0%	24.6%
	Age group	65–74 y	14.0%	18.2%
		75–84 y	19.0%	22.6%
		85–94 y	23.3%	28.6%
		95+ y	26.8%	36.2%
	Care level	1	21.6%	26.4%
		2	19.9%	24.8%
		3	17.7%	18.7%
Dermatologist	Sex	Women	14.7%	16.9%
		Men	19.7%	21.4%
	Age group	65–74 y	13.8%	17.5%
		75–84 y	16.5%	18.0%
		85–94 y	15.8%	17.9%
		95+ y	16.3%	18.7%
	Care level	1	16.6%	18.0%
		2	14.5%	17.9%
		3	12.1%	16.9%
Urologist	Sex	Women	7.5%	10.4%
		Men	34.7%	39.2%
	Age group	65–74 y	16.5%	22.6%
		75–84 y	16.4%	19.6%
		85–94 y	11.8%	14.6%
		95+ y	7.5%	11.0%
	Care level	1	12.5%	14.4%
		2	15.9%	22.0%
		3	19.5%	24.5%
Neurologist/ Psychiatrist	Sex	Women	25.9%	42.0%
		Men	31.7%	46.3%
	Age group	65–74 y	41.3%	55.3%
		75–84 y	37.0%	50.3%
		85–94 y	18.9%	36.6%
		95+ y	7.5%	25.1%
	Care level	1	25.6%	40.9%
		2	30.0%	47.1%
		3	33.6%	48.1%

**Abbreviation:***ENT* Ear, nose and throat

NHR of higher age groups had lesser contacts with most specialists than younger ones with the most pronounced gradient found among neurologists and psychiatrists (55.3% in the youngest vs. 25.1% in the oldest group). Only with ENT specialists a reverse age gradient was found (18.2% vs. 36.2%), for contacts with dermatologists and ophthalmologists no clear age-related pattern appeared.

With a higher care level the proportion of NHR with contacts to specialists decreased. Exceptions with reverse trends were contacts with urologists and neurologists and psychiatrists (Table 3).

The proportion of NHR with dementia seen by a specialist was lower than for those without for almost all specialties (Table 4). The exception were neurologists and psychiatrists (54.9% with dementia vs. 36.0% without). The general patterns were fairly similar before and after institutionalization.

**Table 4:** Proportion of specialist contacts by diagnosis before and after institutionalization (*n* = 18,779)

	Diagnosis	One year before	One year after
Dentist	With dementia	44.5%	50.9%
	Without dementia	44.7%	50.0%
Ophthalmologist	With dementia	34.4%	25.3%
	Without dementia	40.8%	33.7%
	With glaucoma	90.5%	64.6%
	Without glaucoma	29.9%	25.1%
	With diabetes	45.0%	34.7%
	Without diabetes	35.4%	28.7%
Surgeon	With dementia	26.9%	23.4%
	Without dementia	32.1%	28.4%
Gynecologist (women only)	With dementia	8.9%	6.5%
	Without dementia	10.0%	8.0%
ENT specialist	With dementia	19.5%	23.1%
	Without dementia	21.8%	27.2%
Dermatologist	With dementia	15.0%	17.6%
	Without dementia	16.3%	18.1%
Urologist	With dementia	14.7%	15.5%
	Without dementia	13.3%	18.0%
	With prostate hyperplasia	62.5%	52.8%
	Without prostate hyperplasia	9.0%	13.6%
Neurologist / Psychiatrist	With dementia	43.9%	54.9%
	Without dementia	17.5%	36.0%
	With depression	39.5%	50.0%
	Without depression	21.7%	39.8%
	With Parkinson’s disease	72.7%	71.1%
	Without Parkinson’s disease	23.5%	40.6%

**Abbreviation:***ENT* Ear, nose and throat

A smaller proportion of patients with diabetes and glaucoma was seen by an ophthalmologist as well as a smaller proportion of patients with prostate hyperplasia seen by urologists after institutionalization than before. The proportion of patients with Parkinson’s disease seen by a neurologist or psychiatrist was similar while the proportion of patients with depression was higher after institutionalization.

### Predictors for specialist contacts after institutionalization

Strongest predictors for specialist contacts after institutionalization were contacts to a specialist in the year before (Table 5) with an about threefold higher chance for surgeons to an almost ninefold higher chance for urologists. Associated with a higher chance for a contact to most specialist groups after institutionalization were younger age (highest for neurologists and psychiatrists, OR 2.35, lowest for ENT specialists, OR 0.45) and a lower care level (highest for ophthalmologists, OR 2.12) with the exception of urologists. With the exception of neurologists or psychiatrists, NHR with dementia had a lower or equal chance for contacts to most specialties. Sex differences with higher chances for men were only relevant for contacts to dermatologists and urologists.

**Table 5:** Predictors for specialist contacts in the year after institutionalization (odds ratios and 95% confidence intervals; *n* = 18,779)

	Dentist	Ophthalmologist	Surgeon	ENT specialist	Dermatologist	Urologist	Neurologist/Psychiatrist
Men (ref = woman)	1.02 (0.95–1.09)	1.0 (0.92–1.1)	0.85 (0.78–0.92)	0.96 (0.89–1.05)	1.25 (1.15–1.37)	3.24 (2.95–3.55)	1.02 (0.95–1.10)
Age							
65–74 y	1.33 (1.11–1.59)	1.06 (0.86–1.30)	1.25 (1.02–1.55)	0.45 (0.37–0.55)	0.92 (0.73–1.16)	1.66 (1.25–2.22)	2.35 (1.92–2.88)
75–84 y	1.29 (1.09–1.51)	1.07 (0.88–1.29)	1.26 (1.04–1.52)	0.56 (0.47–0.67)	0.93 (0.75–1.14)	1.52 (1.16–2.0)	1.92 (1.60–2.32)
85–94 y	1.09 (0.92–1.28)	1.10 (0.92–1.33)	1.09 (0.90–1.32)	0.72 (0.61–0.86)	0.94 (0.77–1.16)	1.25 (0.96–1.65)	1.44 (1.20–1.73)
95+ y (ref)	1	1	1	1	1	1	1
Care level							
1	1.36 (1.17–1.59)	2.12 (1.73–2.60)	1.82 (1.50–2.22)	1.40 (1.15–1.69)	1.01 (0.83–1.23)	0.60 (0.49–0.74)	0.93 (0.79–1.09)
2	1.27 (1.09–1.49)	1.50 (1.22–1.84)	1.51 (1.23–1.85)	1.34 (1.10–1.63)	1.02 (0.83–1.26)	0.96 (0.78–1.17)	1.10 (0.94–1.30)
3 (ref)	1	1	1	1	1	1	1
Dementia (ref = no dementia)	1.05 (0.98–1.11)	0.73 (0.68–0.79)	0.82 (0.77–0.88)	0.84 (0.78–0.90)	0.98 (0.91–1.07)	0.67 (0.61–0.74)	1.49 (1.39–1.59)
Contact one year before (ref = no contact)	3.08 (2.90–3.27)	5.36 (5.01–5.74)	2.76 (2.59–2.97)	3.60 (3.34–3.88)	4.11 (3.77–4.48)	8.80 (7.96–9.72)	5.20 (4.82–5.61)

**Abbreviation:***ENT* Ear, nose and throat

### Changes in referrals by GPs

Before institutionalization most patients were referred to a specialist by a GP (61.7–73.9%, depending on specialty) while in the year thereafter this proportion decreased significantly (27.8–58.6%) (Table 2). This decrease was lowest for neurologists and psychiatrists (73.9% vs. 58.6%) and much more remarkable for all other specialists. These findings were similar for patients with and without the investigated indicator diagnoses (data not shown).

### Sensitivity analyses

At the end of the second year after institutionalization 3894 persons were still alive, and their data was used for sensitivity analyses. The patterns and magnitudes were similar to the results of the first year. Only the proportion of referrals decreased further (data not shown).

## Discussion

The main finding of this study is that substantial decreases in contact rates with medical specialists for residents after transition to a nursing home are limited to ophthalmologists. A large increase – on the contrary – was found for contacts to neurologists and psychiatrists. The main predictor for a contact to a specialist after admission to a nursing home was the contact prior to admission. A higher age, a higher care level and being diagnosed with dementia was associated with a lower chance of a contact to most specialist groups after admission. Interestingly, the proportion of residents being referred by GPs largely decreased after admission.

With regard to the decrease in contact rates to ophthalmologists this finding might contrast with eye care needs in terms of visual impairments and needs for vision aids that are widespread among NHR [15, 27, 28]. NHR with diagnosed glaucoma or diabetes mellitus are faced with larger absolute reductions in contacts to ophthalmologists than those without, even though ophthalmoscopies are recommended by guidelines annually or biennially [24, 26]. As for contacts to all specialists, a contact prior to the admission to a nursing home was the strongest predictor for contacts thereafter. However, older residents and those diagnosed with dementia had a lower chance for a contact to an ophthalmologist, in line with the international literature and despite known health benefits of adequate eye care in those groups [12]. Possible explanations for this might be low rates of home visits by ophthalmologists and barriers for NHR with mobility constraints to visit ophthalmological practices [20].

On the contrary, contact rates with neurologists and psychiatrists increased after institutionalization by 15.8%. Higher rates than in the general population are known from other studies [2, 3, 19] and highest rates were found among NHR with dementia, Parkinson’s disease and depression. While this increase reflects the disease burden, there are indicators for a remaining undersupply of specialist care for NHR with neurological diseases [14, 29]. Undersupply of medical care is particularly discussed among NHR with depression [30, 31]. In contrast, in the general population the majority of patients with a depression receive medical care primarily provided by GPs [32]. Therefore, our finding that about half of the NHR diagnosed with a depression were seen by a specialist should only cautiously interpreted as a sign of undersupply.

After admission, smaller increases were seen among contacts with dentists, ENT -specialists, dermatologists and urologists. Underlying medical needs might be bad oral health [33], swallowing difficulties associated with malnutrition [5] or incontinence problems [34]. Age gradients were similar before and after institutionalization. For most but not all specialties contact rates were lower the older the NHR were, thus partly confirming results of other studies [3]. Also, lower utilization rates – except for neurologists and psychiatrists - were found among NHR with the highest level of care. A higher level of care is associated with a lesser degree of functional autonomy and discussed as an indicator of the less than optimal resource allocation in medical care for NHR [3, 8]. Specifically in oral health, dental care needs increase and are often not adequately met with a higher level of care dependency [16].

With the known exception of neurologists and psychiatrists [18], consistently lower utilization rates were found among NHR with dementia. Lesser care for NHR with dementia was problematized earlier [3, 8] and this assumption is supported by our findings. Dementia develops over time and its progression is associated with a number of health problems [34]. Dementia and its consecutive care dependency may influence a problematic undersupply of specialist care.

Overall however, our findings do not confirm the assumption of a general underutilization of medical specialists by NHR that is discussed by several authors [3, 18, 22]. Our results rather show a mixed picture of decreases, increases or stable contact rates after institutionalization and they underline the importance of comparing utilization of the same patient during the course of care. The strongest predictor for a specialist contact after admission was the contact to a specialist before. For a part of the residents those contacts are a continuation of care by the same specialist although the data does not contain this information. This might indicate that for those with a chronic health problem the transition to a nursing home per se is not associated with a deterioration of specialist care – at least for most instances. Whether this also applies for NHR with newly occurring health problems remains open. Furthermore, to assess the actual specialist care needs of NHR requires extensive research [35] and has to be interpreted in the context of a particular health system.

Equally, changed therapeutic preferences and goals have to be considered, taking into account multimorbidity and a limited life expectancy of that patient group. Furthermore, disease specific clinical guidelines do not reflect the complexities of old patients with multimorbidities and functional limitations [36]. This might indicate that a palliative approach with a reduction of medication and therapeutic interventions as well as advanced care planning and better compliance with patients´ end-of-life wishes is more adequate for medical care in nursing homes [37]. GPs might approach those medically complex patients differently and put more emphasis on geriatric and palliative aspects, which might reduce the need for medical specialist care in a number of situations. Also, home visits in nursing homes are mainly conducted by GPs [38].

Specialist care needs and criteria for their provision for NHR remain to be discussed in Germany as has been done internationally [39, 40]. However, recommendations in terms of care standards specifically aimed at NHR are not widely established in European countries [41].

In contrast to the findings among other specialties, we found an almost complete coverage of NHR by GPs. This is in line with other studies [3, 8, 18, 19]. However, after institutionalization only the minority of specialist contacts were coordinated through GP referrals. This difference might be only partly explained by a change in health policy in 2013 when a referral fee – that was initially introduced to strengthen GP coordination – was abandoned again, accompanied with an overall reduction in GP referrals. Low rates of referrals initiated by GPs among NHR are seen as an indicator of low structural quality of care [3], for example due to treatment decisions of specialists unknown to the GP that might conflict with other therapeutic goals in multimorbid patients. Further studies should assess how often GPs are aware of specialist contacts and whether they consider problems in cooperation with specialists for the treatment of NHR.

### Strengths and limitations

A major strength of the study is that we could follow a large number of newly admitted NHR over 2 years to gain insight in the utilization of specialist care over time. To our knowledge this is the first study from Germany to have done so. These data are undistorted by recall bias. However, they stem from only one – albeit large – health insurance fund and are therefore not representative of the general population. However, the sociodemographic composition (age, sex, nursing care level) of our study population differs only moderately from the national average of NHR [1].

A general limitation of claims data is that they are collected for reimbursement reasons and not for research. Therefore, information about reasons for or the exact number of contacts within a quarter are unavailable and there might be some uncertainties about the validity of diagnoses.

Questions of structural or process quality as well as patient-oriented outcomes remain open. Also, information about sociodemographic characteristics are very limited and analyses on small geographical scales or on the level of nursing homes are out of scope for this type of data. We could therefore not account for possible regional differences in the availability of medical specialists for nursing homes. Equally, information about GP referrals are limited. They do not contain information about actual communication between specialists and GPs. A referral per se might not guarantee better quality or coordination of care.

## Conclusion

After institutionalization there is no major decrease in contact rates of medical specialists with NHR. The only noticeable exception are lesser contacts to ophthalmologists, including patients with diabetes mellitus, what might contradict recommendations of national guidelines. Especially for NHR with a higher care level or dementia lower chances for contacts to specialists have to be discussed in the context of specific care needs and therapeutic goals. Whether the deescalation of therapeutic goals is more adequate for complex patients in palliative situations and in the context of the particular clinical problem remain open questions. Patients with a very limited life expectancy might benefit from guidelines with a stronger emphasis on multimorbidity. After institutionalization only a minority of specialist contacts occur after referral by GPs, what might contradict their coordination function.

## Data Availability

The data that support the findings of this study were provided by DAK Gesundheit but restrictions apply to the availability of these data, which were used under license for the current study, and so are not publicly available. Data are however available from the authors upon reasonable request and with permission of DAK Gesundheit.

## Abbreviations

GP: General practitioner
ENT: Ear-nose-throat
NHR: Nursing home residents
